# STEMIN- and YAP5SA-induced exosomes prevent cardiomyocyte apoptosis

**DOI:** 10.1016/j.isci.2026.116277

**Published:** 2026-06-22

**Authors:** Adeniyi Adeleye, Siyu Xiao, Ilkin Tetik Altintop, Azeez Muili, Micah Castillo, Preethi Gunaratne, Kacie Waiters, Tasneem Bawa-Khalfe, Bradley K. McConnell, Robert J. Schwartz

**Affiliations:** 1 Department of Biology and Biochemistry, University of Houston, Houston, TX 77204, USA; 2 Sequencing and Editing Core, University of Houston, Houston, TX 77204, USA; 3 Department of Pharmacological and Pharmaceutical Sciences, College of Pharmacy, University of Houston, Houston, TX 77204, USA

**Keywords:** cardiovascular medicine, cell biology

## Abstract

Cardiac regeneration strategies increasingly leverage transient reprogramming to restore function in damaged myocardium. Here, we show that delivery of modRNA encoding STEMIN, a modified serum response factor, together with YAP5SA, a constitutively active YAP1 variant, induces coordinated chromatin remodeling and transcriptional reprogramming in cardiomyocytes. Using ATAC-seq and RNA-seq in rat and human cardiomyocytes, we identify the activation of cell cycle, DNA replication, and survival-associated pathways, alongside a robust induction of microRNAs linked to apoptosis resistance. Exosome profiling reveals selective packaging and release of these miRNAs, which may target key regulators of intrinsic and extrinsic apoptotic pathways, including caspases and TP53-associated signaling. Functional assays demonstrate reduced apoptosis *in vitro* and *in vivo*. These findings support a model in which reprogramming factor-induced exosomal signaling enhances cardiomyocyte survival by inhibiting apoptosis, and highlight a potential therapeutic framework that integrates modRNA delivery with paracrine mechanisms for cardiac repair.

## Highlights

• STEMIN and YAP5SA reprogram cardiomyocytes via chromatin remodeling
• Induce cell cycle, DNA replication, and survival pathways
• Drive exosomal miRNAs targeting apoptosis regulators
• Reduce cardiomyocyte apoptosis in vitro and *in vivo*

## Graphical abstract

**Figure:**
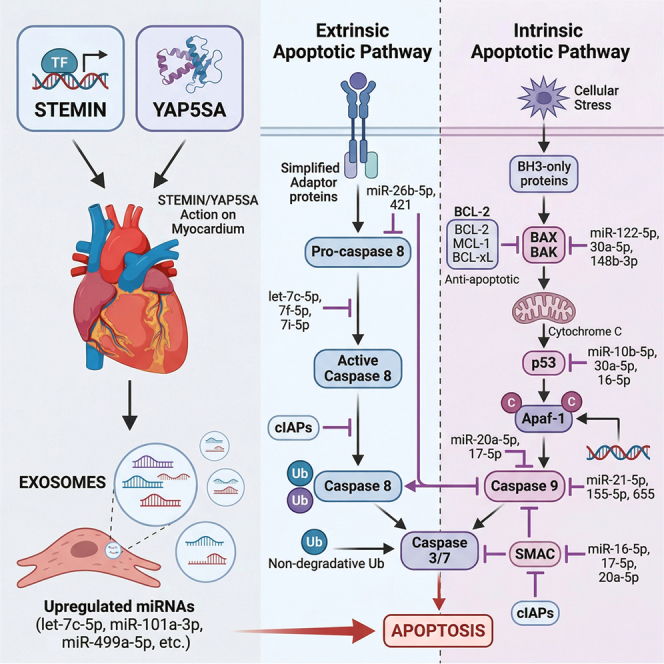

## Introduction

The heart’s ability to regenerate cardiomyocytes declines sharply after early developmental stages,^1^^,^^2^ limiting its capacity for repair after injury. Following a heart attack or myocardial infarction, the heart hardly regenerates lost cells. Instead, the damaged areas are replaced by scar tissue, leading to reduced cardiac function and eventual heart failure.^3^

Most heart attacks are caused by coronary artery disease, which remains the leading cause of death in the U.S. and worldwide.^4^ One of the major consequences of myocardial infarction is cardiomyocyte apoptosis—programmed cell death—driven by the prolonged activation of enzymes called caspases.^5^^,^^6^^,^^7^

Traditionally, it was thought that once the caspase cascade is triggered, it inevitably leads to cell death and contributes to heart failure through the gradual loss of myocytes. Research has shown that caspase-3 activation, in particular, plays a key role in cardiomyocyte loss in heart failure.^8^^,^^9^ Even a low level of apoptosis is enough to impair heart function.^10^

At a modest rate, apoptosis could lead to a progressive loss of contractile myocardium over decades. But in aging or failing hearts, this process accelerates, causing significant muscle loss in just a few months to years.^11^ Excessive cardiomyocyte death weakens the heart’s ability to contract and pump blood, contributing to the progression of heart failure.^6^ Understanding the mechanisms behind cardiomyocyte apoptosis has opened the door to potential therapies aimed at preventing it and preserving heart function.^12^

Other markers of apoptosis include annexin V, DNA fragmentation, and mitochondrial proteins like cytochrome C (CYCS).^8^^,^^13^^,^^14^^,^^15^ For example, CYCS is released from mitochondria during apoptosis, triggering the caspase cascade, while DNA fragmentation is a hallmark of dying cells.^16^^,^^17^

In a notable study, Chang et al.^18^ found that hearts from patients with end-stage heart failure had significantly higher cleaved caspase-3 levels, while those from healthy donors did not. Interestingly, hearts supported by left-ventricular assist devices showed little to no caspase-3 cleavage, suggesting that relieving mechanical stress might reverse some of the apoptotic processes.

One promising approach to heart regeneration involves reprogramming mature cardiomyocytes to a more fetal, proliferative state. This can be achieved using the stem cell reprogramming factors OCT4, KLF4, SOX2, and c-MYC, collectively known as the “OKSM” or Yamanaka factors.^19^^,^^20^ In a previous work, we discovered that a modified version of SRF called STEMIN activates stem cell factors like Nanog and Oct4 in rodent cardiomyocytes, while also suppressing genes responsible for mature cardiac function.^21^^,^^22^

In parallel, we studied YAP5SA—a mutant form of YAP1 that is resistant to inhibition by the HIPPO pathway and promotes cell proliferation.^23^ When combined, STEMIN and YAP5SA induced nuclear replication and cell division in adult heart cells.^21^

While increased proliferation likely plays a major role in heart regeneration, we also observed another important mechanism: the activation of anti-apoptotic microRNAs (miRNAs). Our ATAC-seq data showed that STEMIN and YAP5SA rapidly remodeled the chromatin of ∼100 miRNA genes, many of which were secreted in exosomes. These miRNAs are predicted to target and block key components of both the intrinsic (mitochondrial) and extrinsic (death receptor) apoptosis pathways.

As shown in the graphical abstract, both apoptosis pathways ultimately activate caspases.^24^ Our findings suggest that the miRNAs upregulated by STEMIN and YAP5SA can interfere with this cascade, providing an additional layer of protection against cardiomyocyte loss. Therefore, we hypothesize that exosomes released from STEMIN/YAP5SA-reprogrammed cardiomyocytes carry specific anti-apoptotic miRNAs that protect neighboring myocytes from apoptosis.

## Results

### STEMIN and YAP5SA significantly increase chromatin accessibility to the loci of microRNAs predicted to target key components of the apoptotic pathway, including the caspase and TP53 gene families

**Figure 1:**
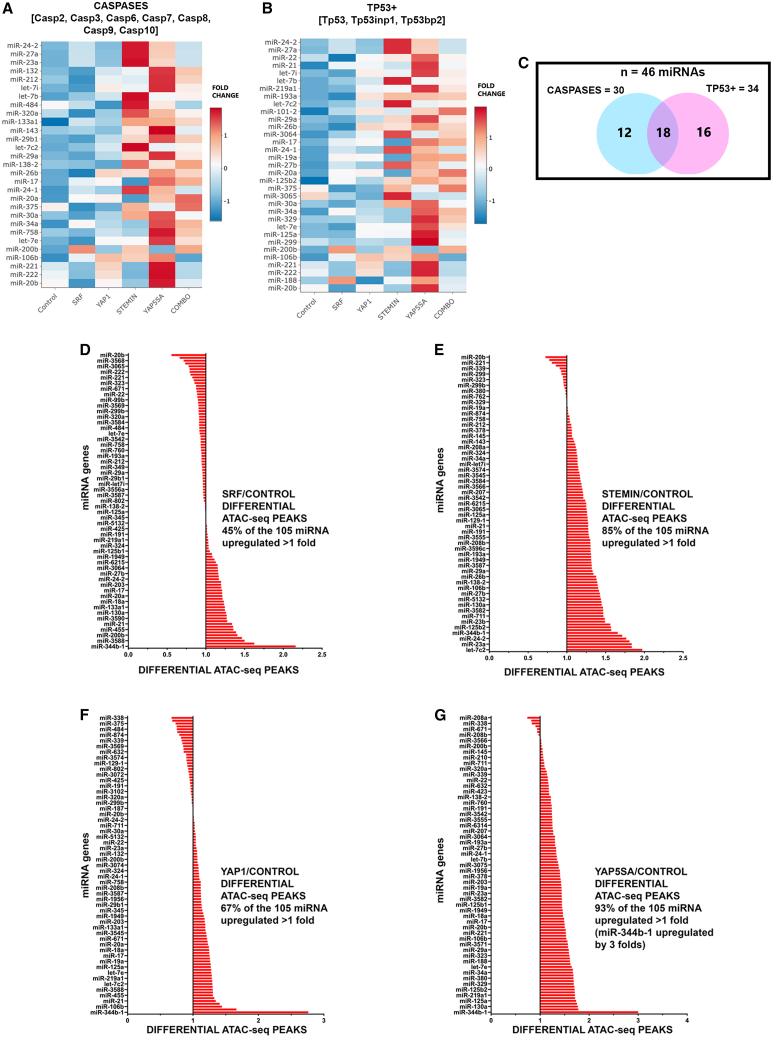
STEMIN and YAP5SA significantly increase chromatin accessibility to the loci of miRNAs predicted to target key components of the apoptotic pathway, including the caspase and *TP53* gene families Heatmaps showing differentially remodeled loci for miRNA genes. Out of 105 miRNA genes considered, STEMIN- and YAP5SA-mRNA enhanced the chromatin accessibility of genes that code for 46 miRNAs. (A–C) that target (A) caspases and ancillary markers, (B) TP53 and ancillary markers, (C) Venn diagram, showing the number of miRNA loci remodeled by STEMIN- and YAP5SA-treated cells, bar charts (D–G) of (D) SRF/control ATAC seq peaks, where 45% of the 105 miRNAs were upregulated by > 1-fold, (E) STEMIN/control ATAC seq PEAKS, where 85% of the 105 miRNAs were upregulated >1-fold, (F) YAP1/control ATAC seq PEAKS, where 67% of the 105 miRNAs were upregulated >1-fold, and (G) YAP5SA/control ATAC seq PEAKS, where 93% of the 105 miRNAs were upregulated >1-fold.

Using ATAC-seq, we analyzed chromatin remodeling in rat cardiac myocytes 24 h after transfection with STEMIN and YAP5SA modRNA.^21^ Compared to their wild-type forms (SRF and YAP1), STEMIN and YAP5SA dramatically enhanced chromatin accessibility at over 100 miRNA gene loci. Specifically, about 85% of 105 miRNAs were upregulated by STEMIN, and 93% of them were upregulated by YAP5SA, with a fold change >1 (Figures 1E and 1G).

These results are consistent with findings from Mercola and colleagues,^25^ who showed that nuclear YAP promotes the expression of many of these same miRNAs in human iPSC-derived cardiomyocytes.

Of particular interest, 46 miRNAs are predicted (by rat-specific miRDB [https://mirdb.org/] and Rat Genome [https://rgd.mcw.edu/rgdweb/homepage/] databases) to target the expression of caspase genes, as well as TP53, and related markers (Figures 1A–1C), including *Casp2*, *Casp3*, *Casp6*, *Casp7*, *Casp8*, *Casp9*, *Casp10*, *Trp53*, *Trp53inp1*, *Trp53i11*, and *Trp53bp2*.

Together, these results show that STEMIN and YAP5SA drive a robust, genome-wide anti-apoptotic gene expression program.

### STEMIN and YAP5SA upregulate anti-apoptotic miRNAs and suppress the expression of apoptotic genes in cardiomyocytes

**Figure 2:**
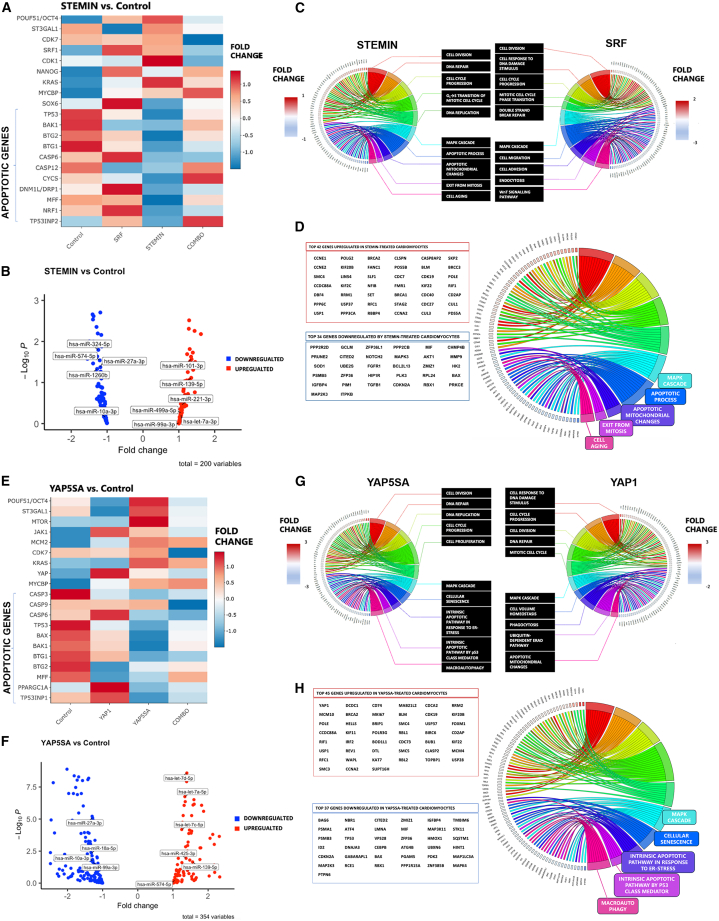
STEMIN and YAP5SA upregulate anti-apoptotic miRNAs and suppress the expression of apoptotic genes in cardiomyocytes OMICS analysis shows. (A) heatmap of differentially expressed apoptotic, cell-cycle arrest, and proliferative markers in STEMIN-treated adult human primary cardiomyocytes. Apoptotic markers, including TP53, CASP6, CASP12, and BAK1, were downregulated. (B) Volcano plot shows STEMIN upregulated miRNAs, including hsa-miR-101-3p and hsa-miR-499a-5p. (C and D) GO chord plots of differentially upregulated and downregulated phenotypes (GO-terms), in STEMIN- vs. SRF-treated cardiomyocytes. (E) A heatmap of YAP5SA-treated adult human primary cardiomyocytes, showing downregulated apoptotic markers, including CASP3, CASP6, CASP9, and TP53. (F) Volcano plot shows differentially expressed miRNAs, including the upregulation of hsa-let-7c-5p, and (G and H) GO chord plots of differentially upregulated and downregulated phenotypes (GO-terms), in YAP5SA- vs. YAP1-treated cardiomyocytes.

To evaluate their global transcriptomic effects, we performed mRNA/miRNA sequencing on total RNA extracted from primary adult human cardiomyocytes transfected with STEMIN and YAP5SA modRNA. Data were visualized using heatmaps, volcano plots (using DESeq2, a *p*-adjusted <0.1), and gene ontology (GO) chord plots via the DAVID GO database, using genes that have a fold change >1 and *p*-adjusted <0.1 (Figures 2A–2D and S1A).

In STEMIN-treated cardiomyocytes (Figure 2A), we observed downregulation of apoptotic and cell-cycle arrest genes, including *SOX6* and *DMNL1* (apoptosis) and *CDKN2A* (cell-cycle arrest). Additional apoptotic genes, such as *TP53*, *CASP6*, *CASP12*, and *BAK1*, were also downregulated. The corresponding volcano plot (Figure 2B) highlights the upregulation of miRNAs *hsa-miR-101-3p* and *hsa-miR-499a-5p*, which are predicted (by human-specific miRDB [https://mirdb.org/] IMOTA [interactive multi omics tissue atlas, [https://ccb-web.cs.uni-saarland.de/imota/mit/]) to target these apoptotic regulators. GO chord plots (Figure 2C) illustrated an enrichment of genes involved in processes that negatively regulate apoptosis in STEMIN vs. SRF controls.

In YAP5SA-treated cardiomyocytes, expression of *CASP3*, *CASP6*, *CASP9*, and *TP53* was reduced (Figure 2E). A volcano plot (Figure 2F) indicated the upregulation of *hsa-let-7c-5p*, another miRNA that may target apoptotic genes. YAP5SA also downregulated *BAX*, *BTG1*, *TP53*, and *CDKN2A* (Figure 2E). In combination treatment, STEMIN + YAP5SA reduced CASP9 expression (Figure 2E).

Across all modRNA-treated groups, several anti-apoptotic miRNAs were significantly upregulated, including *hsa-miR-101-3p*, *hsa-miR-499a-5p*, *hsa-let-7c-5p*, *hsa-let-7a-5p*, *hsa-let-7d-5p*, and *hsa-miR-27a-5p*. These miRNAs likely act as RNAi regulators of the aforementioned apoptotic and cell-cycle arrest genes (Figures 2B and 2F).

GO enrichment analysis revealed that both STEMIN and YAP5SA modRNAs depleted genes involved in biological processes related to apoptosis, mitochondrial apoptotic signaling, mitotic exit, cellular senescence, and intrinsic apoptotic pathways triggered by ER stress or p53-mediated mechanisms, as well as the MAPK cascade. A summary of the differentially-expressed genes by STEMIN and YAP5SA, used to construct the GO chord plots, is provided in Figures 2D and 2H.

To further validate their anti-apoptotic effects, human AC16 cardiomyocytes were cultured and transfected with modRNAs of STEMIN, YAP5SA, their combination (COMBO), and AgomiRs of *hsa-miR-101-3p*, *hsa-miR-499a-5p*, and *hsa-let-7c-5p*. In additional experiments, primary extracted mouse cardiomyocytes were co-transfected with STEMIN or YAP5SA modRNA ± *hsa-miR-499a-5p*. Total RNA was extracted, and qPCR was used to assess the expression of apoptotic and cell death markers. Results are summarized in Figure S3.

Quantitative analysis showed that *CASP3* expression was significantly reduced by 67% (*p* = 0.0029), 84% (*p* = 0.007), and 98% (*p* = 0.002) following treatment with STEMIN, YAP5SA, and COMBO modRNA, respectively. *TP53* expression showed a minimal, non-significant downward (2%) trend with STEMIN (*p* = 0.997), but was more substantially downregulated by 56% (*p* = 0.0135) with YAP5SA and 94% (*p* = 0.007) with COMBO modRNA (Figures S3A and S3B).

Treatment with specific miRNAs also showed a non-significant but downward trend with several apoptotic genes. In *hsa-let-7c-5p*-treated cells, *CASP3* and *CASP9* were reduced by 19% (*p* = 0.3821) and 10% (*p* = 0.6886), respectively. In *hsa-miR-499a-5p*-treated cells, *PDCD4* and *CASP10* were reduced by 16% (*p* = 0.6983) and 30% (*p* = 0.3732), respectively (Figures S3C–S3F).

Furthermore, *hsa-miR-499a-5p* appeared to modestly enhance the anti-apoptotic effects of STEMIN and YAP5SA in *ex vivo* cultured primary mouse cardiomyocytes (Figures S3G–S3I); when combined with YAP5SA, *CASP3* expression was decreased by an additional 7% (*p* = 0.0036), while *TP53* was further reduced by 27% (*p* = 0.0027) when *hsa-miR-499a-5p* was combined with STEMIN.

To validate how hsa-miR-101-3p, hsa-miR-499a-5p, and hsa-let-7c-5p directly target the 3′-UTRs of key apoptotic and anti-survival genes, we cloned the 3′-UTRs of CDKN1A, RUNX1, SOX6, CASP3, RB1, TP53, and PDCD4 downstream of a Firefly luciferase reporter (see Table S1). These constructs were co-transfected with Renilla luciferase into AC16 cardiomyocytes, followed by transfection with the respective AgomiRs. Only canonical seed site miRNA-mRNA interactions were targeted in this experiment; scrambled/deleted-sequence 3′-UTR controls were not used.

Dual luciferase reporter (DLR) assays were performed, and relative Firefly/Renilla luciferase activity was measured (Figure S2). Results showed that the selected miRNAs suppress luciferase expression by binding to their target 3′-UTRs: hsa-let-7c-5p reduced luciferase activity linked to the 3′-UTRs of *TP53* by 66% (*p* = 0.0229), and *RB1* by 50% (*p* = 0.0268) (Figures S2B and S2C). The others suggest a downward trend, though not significant; hsa-let-7c-5p reduced luciferase activity linked to the 3′-UTR of *CASP3* by 50% (*p* = 0.2110) (Figure S2A), hsa-miR-499a-5p decreased *PDCD4* 3′-UTR-linked expression by 10% (*p* = 0.5549) (Figure S2D). hsa-miR-101-3p lowered *SOX6* 3′-UTR-linked expression by 10% (*p* = 0.4679) (Figure S2E).

### Exosomes isolated from STEMIN and YAP5SA-treated cardiac myocytes show the accumulation of anti-apoptotic miRNAs, which may block the expression of apoptotic markers

**Figure 3:**
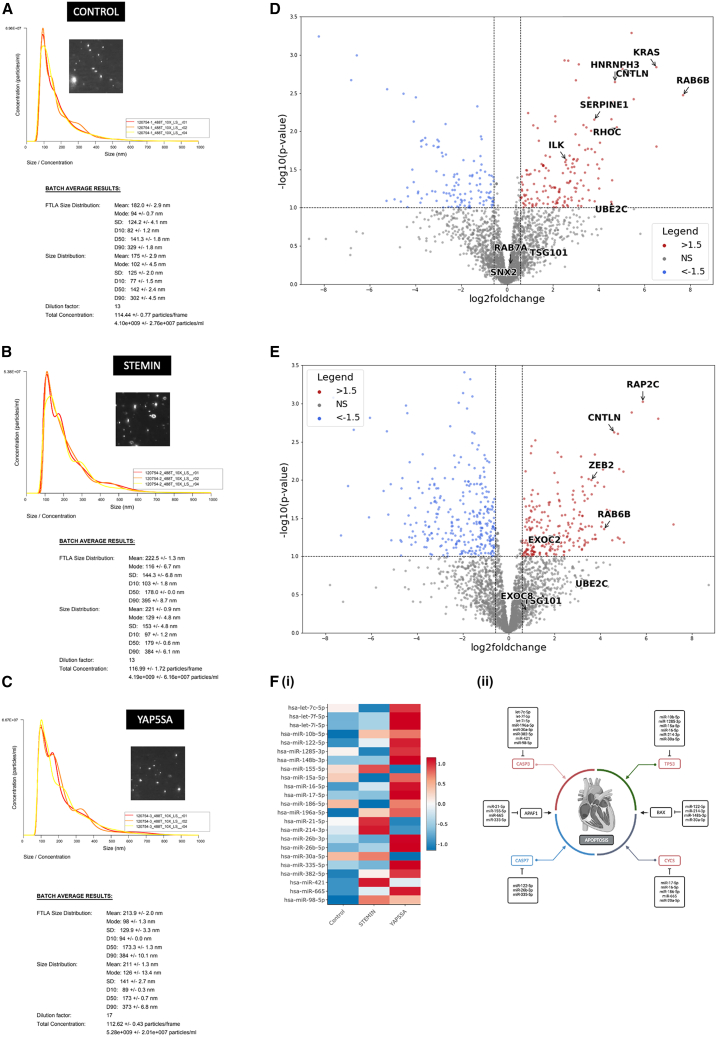
Exosomes isolated from STEMIN and YAP5SA-treated cardiac myocytes show the accumulation of anti-apoptotic miRNAs, which may block the expression of apoptotic markers Nanoparticle tracking analysis of exosomes secreted into culture media from AC16 cardiomyocytes transfected with (A–C), (A) Empty reagent (control), (B) STEMIN-mRNA, (C) YAP5SA-mRNA. Duplicates of ∼10 μg of secreted exosomes/sample were identified by an Orbitrap mass spectrophotometer, and following proteomic analysis, volcano plots of duplicate samples are shown for (D) STEMIN, with preferentially secreted proteins such as KRAS, RAB6B, RHOC, and HNRNPH3. (E) YAP5SA, with preferentially secreted proteins such as UBE2V2, ZEB2, RAP2C, SRM, and THYN1; they both share serpin, CNTLN1, and CTSC. OMICS analysis shows a heatmap from miR-seq of (Fi) control-, STEMIN- and YAP5SA-generated exosomes, and (Fii) differentially expressed anti-apoptotic miRNAs, and their predicted apoptotic targets determined by specific miRDB [https://mirdb.org/] and IMOTA (interactive multi-omics tissue atlas [https://ccb-web.cs.uni-saarland.de/imota/mit/]).

To assess their induced exosome production, AC16 cardiomyocytes were stabilized in culture and then transfected with control (empty reagent), STEMIN, or YAP5SA modRNAs. Exosomes were isolated from the culture media using the Exo-Easy Maxi Kit (QIAGEN) and characterized by nanoparticle tracking (Figures 3A–3C); all groups produced exosomes within the expected size range. Their miRNA cargoes were also determined using miRNA-seq and analyzed into a heatmap using DESeq2 at a fold change >1 and *p*-adjusted <0.1.

Protein content from exosome samples was first assessed by silver-stained SDS-PAGE (Figure S4), showing no dominant bands. Proteins from duplicate exosome samples were identified by Orbitrap mass spectrometry and analyzed using the Mascot search engine and gpGrouper. Proteomic analysis revealed distinct protein signatures: STEMIN exosomes (Figure 3D) were enriched in KRAS, RAB6B, RHOC, and HNRNPH3. YAP5SA exosomes (Figure 3E) contained UBE2V2, ZEB2, RAP2C, and THYN1. Shared proteins included α-synuclein, serpine, CNTLN1, and exosome markers like tetraspanins, Rab proteins, and TSG101.

miRNA sequencing revealed that YAP5SA exosomes were enriched in the let-7 family miRNAs, while STEMIN exosomes showed higher levels of miR-21-5p, miR-214-3p, and miR-421 (Figure 3Fi). Collectively, exosomes from both groups contained miRNAs such as hsa-let-7c-5p, hsa-miR-10b-5p, miR-122-5p, miR-15a-5p, miR-16-5p, miR-21-5p, and miR-98-5p, among others. Many of these miRNAs are predicted to target pro-apoptotic genes, including CASP3, CASP7, TP53, BAX, CYCS, and APAF1 (Figure 3Fii), using the human-specific miRDB (https://mirdb.org/) and IMOTA (interactive multi-omics tissue atlas, (https://ccb-web.cs.uni-saarland.de/imota/mit/) databases.

Comparing the miRNA profiles to known exosomal sequence motifs (GGAG, UGAG/UCAG, CCCU, and UCCU), we found that 17 of the 23 identified miRNAs (∼70%) contained at least one of these motifs (Table S2), making our samples consistent with known EXO-motifs.

### STEMIN, YAP5SA, and their upregulated miRNAs suppress apoptosis in cardiomyocytes

**Figure 4:**
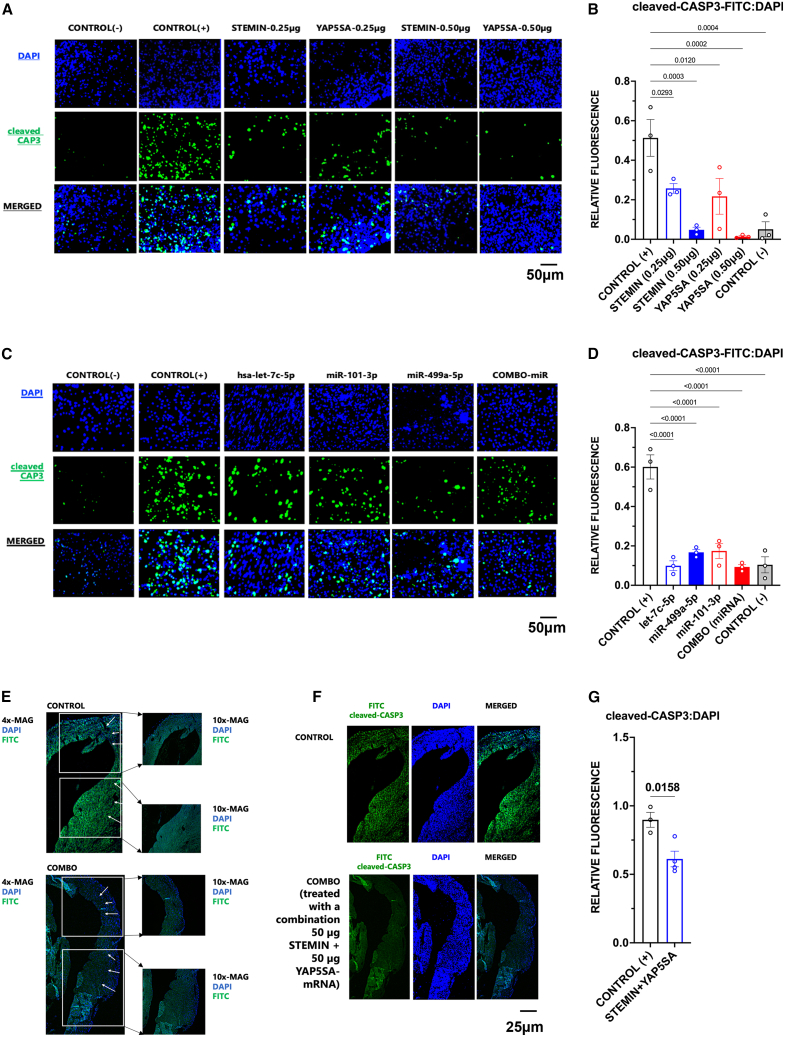
STEMIN, YAP5SA, and their activated miRNAs block staurosporine-induced apoptosis in cardiomyocytes (A) Immunofluorescence images of STEMIN- and YAP5SA-treated cardiomyocytes. (B) Quantitative cleaved-CASP3 (FITC)/DAPI relative fluorescence bar chart from STEMIN- and YAP5SA-treated cardiomyocytes. (C) Immunofluorescence images of hsa-let-7c-5p-, miR-101-3p-, and miR-499a-5p-treated cardiomyocytes. (D) Quantitative cleaved-CASP3 (FITC)/DAPI relative fluorescence bar chart from hsa-let-7c-5p-, miR-101-3p-, and miR-499a-5p-treated cardiomyocytes. Results of assays in (A–D) were performed in triplicate (*n* = 3); data are represented as mean ± SEM, and analyzed by one-way ANOVA. (E–G) Immunohistochemistry images and plot, showing 4× and 10× magnified images of cleaved-CASP3 immuno-stained tissue sections of infarcted mice hearts injected with an empty reagent and an equimolar combination (COMBO) of STEMIN + YAP5SA (white arrows showing point of injection), (F) split channel images and (G) bar chart showing the quantitative cleaved-CASP3/FITC:DAPI mean relative fluorescence. Results of assays in (E–G) were performed on seven animals: three control mice (two males, one female), four treatment mice (two males, two females). Data are represented as mean ± SEM, and analyzed by Welch test. Top and bottom vertical error bars and horizontal bars with p-values are included in the bar graphs 4B, 4D, and 4G.

To evaluate whether STEMIN, YAP5SA, and their associated miRNAs can reduce apoptosis, human AC16 cardiomyocytes were transfected with two doses (0.25 μg and 0.50 μg) of STEMIN and YAP5SA modRNAs, or with AgomiRs of hsa-miR-101-3p, hsa-miR-499a-5p, and hsa-let-7c-5p. The cells were then treated with staurosporine, a known apoptosis inducer. After treatment, cells were fixed, permeabilized, and stained for cleaved caspase-3, followed by immunofluorescence imaging (Figures 4A–4D).

In parallel, a second experiment used flow cytometry with CellEvent caspase-3/7 detection reagent to quantify apoptotic events (Figures S5A–S5L). (SSC-A, or side scatter-area, is a parameter used to measure the intensity of light scattered to the side when cells pass through the laser beam in a flow cytometer, and it is quantified in arbitrary units [a.u.s]. Apoptosis was estimated via SSC-A and CellEvent caspase 3/7 Fluorescence [FITC], and analyzed with Floreada [https://floreada.io/]).

Immunofluorescence results (cleaved-CASP3 normalized with DAPI) showed that both modRNAs decreased apoptosis: STEMIN reduced apoptosis by 92% (*p* = 0.0003) at 0.50 μg and 50% (*p* = 0.0293) at 0.25 μg. YAP5SA reduced apoptosis by 98% (*p* = 0.0002) at 0.50 μg and 58% (*p* = 0.012) at 0.25 μg. miRNAs also showed significant effects, hsa-let-7c-5p, 98% reduction; hsa-miR-101-3p, 72% reduction; and hsa-miR-499a-5p, 73% reduction (all three at a *p* < 0.0001).

Flow cytometry data confirmed these findings, albeit more conservatively: STEMIN, YAP5SA, and COMBO (STEMIN+YAP5SA) modestly reduced apoptosis by 37%, 24%, and 37%, respectively (Figures S5A–S5E). hsa-let-7c-5p, 16% reduction; hsa-miR-101-3p, 33% reduction; hsa-miR-499a-5p, 34% reduction; and miRNA combo (COMBO), 33% reduction (Figures S5F–S5I). The modest result in the flow cytometry result compared to the immunofluorescence result may be due to the higher sensitivity of the cleaved-CASP3 antibody used, and also the low sensitivity of the flow cytometer equipment and the CellEvent CASP3/7 kit.

These results suggest that both modRNAs and their downstream miRNAs mitigate apoptosis in cardiomyocytes exposed to staurosporine.

To test the anti-apoptotic effects of STEMIN and YAP5SA *in vivo*, seven adult mice (control group, two males, one female; treatment group, two males, two females) underwent myocardial infarction via left coronary artery (LCA) ligation. Five minutes after ligation, the left ventricles of the treatment group were injected with a combined modRNA treatment (50 mg of STEMIN +50 mg of YAP5SA).

After 48 h, heart tissues were collected and analyzed using immunohistochemistry to detect cleaved caspase-3, a marker of apoptosis. The results, shown in Figures 4E–4G, revealed that the COMBO-modRNA treatment reduced apoptosis by approximately 28% (*p* = 0.0158) compared to the control.

These findings demonstrate that a single injection of STEMIN and YAP5SA rapidly and significantly protects cardiac muscle cells from apoptosis following a heart attack in mice. This study does not distinguish cell-autonomous effects from paracrine exosome effects, but takes both effects as a whole.

### Secreted exosomes from STEMIN and YAP5SA modRNA-transfected cardiac myocytes, transferred to staurosporine-treated cardiomyocytes, hinder the expression of apoptotic markers

To test whether exosomes from STEMIN- and YAP5SA-transfected cells can protect against apoptosis, AC16 cardiomyocytes were treated with exosomes derived from control (AC16-only), STEMIN-, or YAP5SA-treated cells. After staurosporine exposure to induce apoptosis, cells were stained with CellEvent caspase-3/7 detection reagent and analyzed via flow cytometry (Figures S5J–S5L).

**Figure 5:**
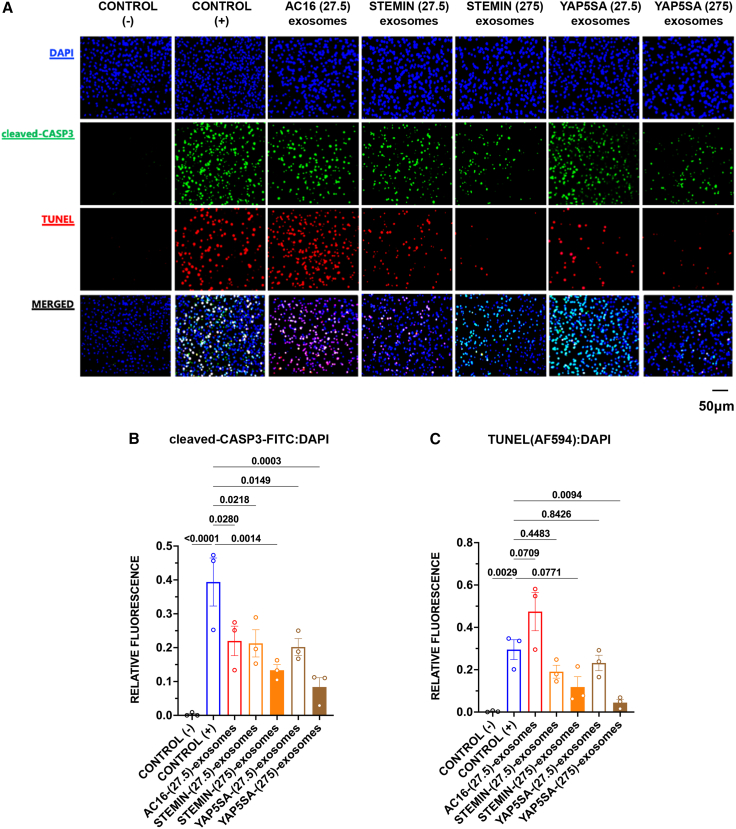
Secreted exosomes from STEMIN and YAP5SA modRNA-transfected cardiac myocytes, transferred to staurosporine-treated cardiomyocytes, hinder the expression of apoptotic markers (A–C) Immunohistochemistry images and plot, showing images of cleaved-CASP3 and TUNEL immuno-staining of fixed and permeabilized AC16 cells previously cultured with exosomes from (A) AC16-only cells, STEMIN-treated AC16 cells, and YAP5SA-treated AC16 cells, after exposure to staurosporine: split channel images of DAPI (blue), cleaved-CASP3 (green), and TUNEL (red), and (B and C) bar chart showing (B) quantitative cleaved-CASP3/FITC:DAPI and (C) TUNEL:DAPI mean relative fluorescence. Data are represented as mean ± SEM and analyzed by one-way ANOVA. Top and bottom vertical error bars and horizontal bars with p-values are included in the bar graphs 5B and 5C.

In a parallel experiment, cells were exposed to varying doses (1×, with 27.5 ng total protein and 10×, with 275 ng total protein) of STEMIN- or YAP5SA-derived exosomes, exposed to staurosporine to induce apoptosis, then fixed and stained for cleaved caspase-3 and TUNEL (terminal deoxynucleotidyl transferase dUTP nick end labeling, a method for detecting DNA fragmentation by labeling the 3′- hydroxyl termini in the double-strand DNA breaks generated during apoptosis). Immunofluorescence images were analyzed to assess apoptotic activity (Figures 5A–5C).

Flow cytometry results showed that STEMIN-derived exosomes reduced apoptosis by 37% and YAP5SA-derived exosomes reduced apoptosis by 38% (Figures S5K and S5L).

Immunofluorescence assays confirmed these findings: STEMIN exosomes decreased caspase-3 cleavage by 46% (*p* = 0.0218) at 1× dose, 66% (*p* = 0.0014) at 10× dose, and reduced DNA fragmentation by 34%, though not so significantly (*p* = 0.4483) at 1× dose and 62% (*p* = 0.0771) at 10× dose. YAP5SA exosomes decreased caspase-3 cleavage by 49% (*p* = 0.0149) at 1× dose, 80% (*p* = 0.0003) at 10× dose, and reduced DNA fragmentation by 20%, though not so significantly (*p* = 0.8426) at 1× dose and 80% (*p* = 0.0094) at 10× dose.

Together, these results suggest that exosomes from STEMIN and YAP5SA-treated cells may confer protection against apoptosis in cardiomyocytes.

## Discussion

Our study demonstrates that STEMIN and YAP5SA are associated with reduced apoptosis in cardiomyocytes by upregulating pro-survival miRNAs that are predicted to target apoptotic and anti-viability genes. This mechanism—blocking apoptosis—likely underlies the enhanced survival of cardiomyocytes observed with these factors.

Using ATAC-seq, a chromatin accessibility assay, we identified over 100 genes encoding miRNAs predicted to suppress apoptosis; their chromatin loci became more accessible after STEMIN or YAP5SA transfection, suggesting a role in chromatin remodeling and potentially blocking anti-apoptotic effects. However, the miRNAs may also have other non-canonical roles. Importantly, STEMIN and YAP5SA induced both overlapping and distinct sets of miRNAs, suggesting possible complementary and unique effects.

Some of these miRNAs were also secreted in exosomes, which are capable of delivering these protective signals to neighboring cells via paracrine signaling. Using dual-luciferase assays, we validated that some of the miRNAs, including let-7c-5p, significantly targeted the 3′-UTRs of known apoptotic and anti-proliferative genes like TP53 and RB1, while others showed downward trends, requiring further validation. Results from a functional assay, like immunofluorescence, showed the anti-apoptotic activity of the miRNAs, their exosomes, and the parent modRNAs *in vitro*, while the flow cytometry results showed a modest reduction in apoptosis.

Notably, STEMIN and YAP5SA curtailed caspase-3 activation by at least 38% in infarcted murine hearts (from four animals treated with STEMIN+YAP5SA, vs. three that were untreated); particularly, the reduction in apoptosis/cleaved-CASP3 staining was observed in the region near the point of injection. This mirrors results in cultured cardiomyocytes treated with apoptotic toxins.

This rapid suppression of apoptosis, alongside proliferative activation, could offer a dual mechanism for cardiac regeneration. Moreover, exosomal miRNAs may serve as a paracrine defense system, spreading protection throughout the heart after localized modRNA injection.

Exosomes from STEMIN- and YAP5SA-treated cells were further analyzed using nanoparticle tracking and exo-miR-seq, confirming their size and miRNA enrichment. With an increase in (biochemical and electrophysiological) validation, sample size, long-term studies, and data with stronger statistical strength in both *in vivo*, *in vitro*, and *ex vivo* models, exosomes could prove to be an efficient route for intercellular communication, with implications for regenerative medicine, drug delivery, and disease diagnostics.^26^^,^^27^^,^^28^^,^^29^

### Limitations of the study

For this study, we focused on the biochemical observation of apoptosis rather than cardio-physiological effects. We also did not consider the contribution of cardiomyocyte proliferation to the net myocyte cell loss.

## Resource availability

### Lead contact

• Requests for further information and resources should be directed to and will be fulfilled by the lead contact, Robert J. Schwartz at sjrobert@central.uh.edu.

### Materials availability

• This study did not generate new unique reagents.
• pcDNA and modRNA (modified RNAs) of STEMIN and YAP5SA are available upon contacting Robert J. Schwartz at sjrobert@central.uh.edu.

### Data and code availability

• Data reported in this study will be shared by the lead contact upon request.
• This study does not report any original code.
• Any additional information required to reanalyze the data reported in this study is available from the lead contact upon request.

## Acknowledgments

Financial support and sponsorship: Research support for this study to R.J.S. was through the 10.13039/100007144University of Houston, a Hugh Roy and Lillian Cranz Cullen Distinguished University Professorship, a Cullen Endowed Chair, the Texas Board of Higher Education, and a Sponsored Research Agreement from Animatus Biosciences, 10.13039/100023970LLC.

Power analysis of immunohistochemistry data from *in vivo* studies (Figures 4E–4G) was conducted by Dr. Moosa Tatar (University of Houston).

## Author contributions

Conceptualization, R.J.S. and A.A.; methodology, A.A.; validation, A.A. and R.J.S.; formal data analysis, A.A., R.J.S., M.C., P.G., K.W., and T.B.-K.; resources, A.A., R.J.S., B.K.M., A.M., and I.T.A.; writing – original draft preparation, review, and editing, A.A., and R.J.S.; visualization, A.A., M.C.; and funding acquisition, R.J.S. All authors contributed to the article and approved the submitted version.

## Declaration of interests

R.J.S. is a cofounder of Animatus Biosciences, and he received research funds from the company.A.A. and R.J.S. have a patent application related to this work. Other authors declared that there are no conflicts of interest. This research and patents were underwritten in part by a grant from Animatus Biosciences LLC, in which there is a financial interest. An acceptable management plan, reviewed and recommended by the University of Houston Conflict of Interest Committee, was designed to address any conflict of interest.

## Declaration of generative AI and AI-assisted technologies in the writing process

During the preparation of this work, the lead author, A.A., used Genspark.ai to generate the Graphical Abstract. After using the tool, he reviewed and edited the content as needed and takes full responsibility for the publication’s content.

## STAR★Methods

### Key resources table

**Table:** 

REAGENT or RESOURCE	SOURCE	IDENTIFIER
**Antibodies**		

Cleaved-CASP3	Cell Signaling Technology (Asp175, 5A1E, Rabbit Monoclonal Antibody)	Cat #9661; RRID: AB_2341188
Alexa Fluor 488	Invitrogen	Cat #A-11008

**Biological samples**		

Luciferase-pcDNA3	Addgene	Cat #18964

**Chemicals, peptides, and recombinant proteins**		

Staurosporine	MedChem Express	Cat #HY15141

**Critical commercial assays**		

Exo-easy Maxi kit	Qiagen	Cat #76064
Zymo Quick-RNA kit	Zymo Research	Cat #R1054
miRNeasy kit	Qiagen	Cat #217084
qScript cDNA SuperMix	QuantaBio	Cat #95048-100
Poly(A) polymerase and ATP	New England Biolabs	Cat #M0276S
SYBR Green Master Mix	Applied Biosystems™	Cat #A25742
HiScribe® T7 ARCA mRNA kit (with tailing)	New England Biolabs	Cat #E2060S
Lipofectamine MessengerMAX	ThermoFisher	Cat #LMRNA008
Lipofectamine RNAiMAX	ThermoFisher	Cat #13778075
jetPRIME® transfection reagent	PolyPlus Transfection SA	Cat #114-07
TUNEL Assay Kit with Alexa Fluor 594	Invitrogen	Cat #10246
Prolong Diamond Anti-Fade Mounting Medium with DAPI	Invitrogen	Cat #P36962
Human Cardiomyocyte Extra-cellular Matrix - 6 Well Plates	Celprogen	Cat #E36044-15-6Well
Human Cardiomyocyte Serum Free Media	Celprogen	Cat #M36044-15
Human Cardiomyocyte Complete Media with Serum	Celprogen	Cat #M36044-15S
DMEM/F12 medium	Gibco	Cat #11320033
Passive Lysis 5X Buffer	Promega	Cat #E1910
Dual-Glo® Luciferase Assay System	Promega	Cat #E2920

**Experimental models: Cell lines**		

AC16 Cardiomyocytes	Sigma Aldrich	Cat #SCC109
Human Cardiomyocyte Cell Culture (Frozen Vial)	Celprogen	Cat #36044-15

**Oligonucleotides**		

hsa-miR-499a-5p miRNA AgomiR	Applied Biological Materials, Inc.	Cat #MAH02779
hsa-miR-101-3p miRNA AgomiR	Applied Biological Materials, Inc.	Cat #MAH01031
hsa-let-7c-5p miRNA AgomiR	Applied Biological Materials, Inc.	Cat #MAH01009

**Recombinant DNA**		

pcDNA-5’-UTR-TNNT2-STEMIN	Schwartz Lab	
pcDNA-5’-UTR-TNNT2-YAP5SA	Schwartz Lab	

**Software and algorithms**		

Fiji/ImageJ	National Institutes of Health	
Snap Gene	Siemens	
DeSeq2 (R-studio)	Bioconductor	

### Experimental model and study participant details

#### Cells and culture conditions

Primary human cardiomyocytes (Celprogen) were cultured in both serum-containing (Media with Serum Cat# M36044-15S) and serum-free media (Serum-free media Cat# M36044-15), supplemented with an extracellular expansion matrix (Extracellular Matrix Cat# E36044-15) (all reagents from Celprogen). Primary human cardiomyocytes were cultured in serum-containing media to normalize them, and then gradually weaned off serum (50% - 0% within 4 days) according to the manufacturer’s protocol. These cardiomyocytes were used for mRNA/miRNAseq.

AC16 cardiomyocytes (Sigma Aldrich) were also utilized and maintained in standard culture conditions: DMEM/F-12 medium (Gibco) supplemented with 12.5% fetal bovine serum (FBS) and 1% Penicillin-Streptomycin. AC16 cells passaged 3-6 times were used for experiments such as qPCR, Dual Luciferase Reporter assays, and immunoassays. AC16 cardiomyocytes used for exosome experiments were grown under serum-free conditions.

Both primary human cardiomyocytes (Celprogen) and AC16 cells (Sigma Aldrich) were validated by microscopy (showing adherent, flattened, cobble-stone like morphology, growing in a monolayer). No mycoplasma testing was done, but contamination was not observed throughout the experiments.

#### Animals and myocardial infarction model

Seven adult (8-10 weeks) C57BL/6J mice (Control Group: 2 males, 1 female, Treatment Group: 2 males, 2 females), 15-20g in weight, were maintained according to the *Guide for the Care and Use of Laboratory Animals* (NIH, 8th edition) under IACUC protocols 15-055 and 16-015 (University of Houston).

Myocardial infarction was induced in the mice by left coronary artery (LCA) ligation for 4 hours, and then their left ventricular myocardium was injected with COMBO – 50 μg each of STEMIN and YAP5SA modRNAs (total 100 μg), mixed with Lipofectamine MessengerMAX in 60 μL volume (Control Group mice were injected with empty Lipofectamine), 5 minutes post-ligation during open-chest surgery. After 48 hours, the animals were sacrificed, and their hearts were harvested and stored.

The survival rate for this surgery is 50%. Starting with 14 animals, only 7 animals survived the procedure and were used for subsequent assays.

Statistical analysis revealed no significant influence or association of sex on cardiomyocyte apoptosis levels, and no sex-specific effects were observed across experimental groups.

### Method details

#### Methods

##### STEMIN and YAP5SA significantly increase chromatin accessibility to the loci of microRNAs (miRNAs) predicted to target key components of the apoptotic pathway, including the caspase and *TP53* gene families

Rat neonatal cardiomyocytes were transfected with synthetic modRNA encoding SRF, YAP1, STEMIN, YAP5SA, and COMBO (COMBO-modRNA contains equimolar amounts of STEMIN- and YAP5SA-modRNA) with Lipofectamine MAX. Controls were transfected only with Lipofectamine MAX. After 24 hrs., cardiomyocytes were prepared and submitted for ATAC-sequencing (Active Motif).

##### STEMIN and YAP5SA upregulate anti-apoptotic miRNAs and suppress the expression of apoptotic genes in cardiomyocytes

Primary adult human cardiomyocytes were cultured and stabilized for 24 hours, using regular media, after which they were serially serum-starved for 72 hours, using serum-free media, to achieve a steady state and terminally mature conditions. The resulting cells were then transfected twice within 48 hours with the following: empty reagent (control), 2.5 μg mRNA of wild-type-SRF, STEMIN (mutant-SRF), wild-type-YAP1, YAP5SA (mutant-YAP1), and COMBO (1.25 μg mRNA of STEMIN + 1.25 μg mRNA of YAP5SA), six conditions in all. Total cellular RNA was prepared from the cells, and miRNA/mRNA-seq was done on them. All assays were done in triplicate (n = 3).

##### Exosomes isolated from STEMIN and YAP5SA-treated cardiac myocytes show the accumulation of anti-apoptotic miRNAs, which may block the expression of apoptotic markers

miRNAseq was done on the exosomes purified from the previously-collected cell-free, serum-free conditioned media used in culturing AC16 cells transfected with empty reagent (control) and mRNAs of STEMIN and YAP5SA.

##### STEMIN, YAP5SA, and their activated miRNAs block staurosporine-induced apoptosis in cardiomyocytes

AC16, which was blunted by prior transfections with synthetic STEMIN and YAP5SA modRNA at 0.50 μg and 0.25 μg, and AgomiRs hsa-let-7c-5p, hsa-miR-101-3p, hsa-miR-499a-3p, and COMBO-miRNA. Afterwards, all the cells were fixed and permeabilized with 4% PFA (paraformaldehyde) and 0.3% Triton-X-100, and then immuno-stained with cleaved-caspase-3 primary antibody and Alexa Fluor™ 488 fluorescent conjugate and mounted with mounting medium containing DAPI. The slides were cured for 48 hours and imaged by immunofluorescence. Assays were done in triplicate (n = 3) and analyzed by one-way ANOVA.

Myocardial infarction was induced in adult mice by left coronary artery (LCA) ligation for 4 hours, and then their hearts’ left ventricles were injected with empty reagent (control) and 100 μg of COMBO-mRNA (50 μg STEMIN-mRNA + 50 μg YAP5SA-mRNA). They were kept alive for 48 hours, after which the animals were sacrificed, and their hearts harvested and stored. The hearts were then fixed in paraffin wax, and 5-10 μm tissue sections from them were obtained on glass slides; these sections were deparaffinized, immuno-stained with cleaved-caspase-3 primary antibody plus Alexa Fluor™ 488 fluorescent conjugate, and mounted with mounting medium containing DAPI. The slides were cured for 48 hours, and immunofluorescence images were obtained. The images were then analyzed with ImageJ software, and the resulting data were organized into relative fluorescence plots.

##### Secreted exosomes from STEMIN and YAP5SA modRNA-transfected cardiac myocytes, transferred to staurosporine-treated cardiomyocytes, hinder the expression of apoptotic markers

AC16 cardio-myocytes were cultured in regular media for 24 hours in a 3-well removable glass chamber after which the media was refreshed with serum-free media plus AC16-only-, STEMIN-generated and YAP5SA-generated exosomes (the exosomes were normalized using the total amount of exosomal protein extract, 275ng of protein for the AC16-only-exosomes group, 27.5ng of protein for the 1x STEMIN-exosome group, 275ng of protein for the 10x STEMIN-exosome group, 27.5ng of protein for the 1x YAP5SA-exosome group and 275ng of protein for the 10x YAP5SA-exosome group). After another 12 hours, the cells were treated with 2μM staurosporine to induce apoptosis for 6 hours. Afterwards, the cells were fixed and permeabilized with 4% PFA (paraformaldehyde) and 3% Triton-X-100, and then immuno-stained in a TUNEL assay plus Alexa Fluor™ 594 fluorescent conjugate, and separately with cleaved-caspase-3 primary antibody plus Alexa Fluor™ 488 fluorescent conjugate. They were then mounted with DAPI-containing mounting medium. The glass slides were cured for 48 hours, and immunofluorescence images were obtained. Assays were done in triplicate (n = 3) and analyzed by one-way ANOVA.

##### Selected miRs hsa-miR-101-3p, hsa-miR-499a-5p, and hsa-let-7c-5p target the 3’-UTRs of apoptotic and anti-survival markers

1.0 μg FIRE-3’-UTRs were co-transfected with 1.0 μg Renilla luciferase expression construct into AC16 cardiomyocytes previously cultured and stabilized in regular media. After 4 hours, the media was refreshed, and the cells were transfected with empty reagent (control), 1.0 μg AgomiRs of hsa-miR-101-3p, hsa-miR-499a-5p, and hsa-let-7c-5p. Assays were done in triplicate (n = 3) and analyzed by the Welch Test.

##### STEMIN, YAP5SA, and their induced anti-apoptotic microRNAs in cardiomyocytes downregulate apoptotic gene activity

AC16 cardiomyocytes were stabilized in regular media for 24 hours, and then transfected with an empty transfection reagent as a control. All assays were done in triplicate (n = 3) and analyzed by one-way ANOVA for multiple comparisons (Figures S3A, S3B, and S3G–S3I) and Welch Test for single comparisons (Figures S3C–S3F).

##### Flow cytometric estimation of apoptosis in modRNA, miRNA, and exosome-treated cardiomyocytes

AC16 cardiomyocytes were cultured and stabilized in regular media for 24 hours and then transfected with modRNA and miRNAs. In parallel, AC16 cardiomyocytes were cultured and stabilized in regular media for 24 hours, refreshed with serum-free media, and then transfected (exofected) with 10x-AC16-only-exosomes (control), 10x-STEMIN- and 10x-YAP5SA-generated exosomes, containing 275ng total exosomal protein extract (exosomes were normalized by the amount of total exosomal protein extract estimated from a Silver Staining assay). Two control groups were also made for each experiment: one positive control and one negative control, both transfected with empty reagent. After 24 hours, the cells were charged with 2μM staurosporine to induce apoptosis for six hours (the positive control was treated similarly with staurosporine, while the negative control was untouched). The cells were then collected, 100,000 of them were strained through 35 μm nylon mesh screens into 12x75 mm flowtubes™, stained with CellEvent Caspase 3/7 green detection reagent to detect cells undergoing apoptosis, via flow cytometry, using SSC-A. SSC-A, or Side Scatter - Area, is a parameter used to measure the intensity of light scattered to the side when cells pass through the laser beam in a flow cytometer, and it is quantified in arbitrary units (a.u.). Apoptosis was estimated using the Cell Event Caspase 3/7 Fluorescence (FITC) and analyzed with Floreada (https://floreada.io/) (Figures S5A–S5L).

#### mRNA synthesis

Modified messenger RNAs (modRNAs) were synthesized by linearizing plasmids containing the desired mutations using *HindIII*, followed by *in vitro* transcription with T7 RNA polymerase and ARCA/NTP mix (no modified nucleotides were used, only GTP, CTP, UTP, and ATP).

Transcription and ARCA capping were performed at 37°C overnight. Lithium chloride (LiCl) precipitation at −20°C for 12 hours or overnight was used to remove unincorporated nucleotides, proteins, and DNA templates. From experimental experience, a longer period increased yields and purity. The resulting capped RNA was polyadenylated using Poly(A) polymerase (HiScribe® T7 ARCA mRNA kit with tailing, New England Biolabs) and purified with the RNA Clean & Concentrator kit (Zymo Research). RNA quality was assessed via NanoDrop spectrophotometry. Expression of the modRNAs was verified by transfecting them into AC16 cells and doing Western blot and qPCR.

#### ATAC-seq and analysis

Rat neonatal cardiomyocytes were harvested 24 hours after treatment of mRNAs Srf, Yap1 modRNA, STEMIN, YAP5SA, and a combination of STEMIN+YAP5SA delivered by Lipofectamine MessengerMAX (ThermoFisher). Approximately 300,000 cells per sample were submitted for sequencing. Transfected neonatal cardiomyocytes were centrifuged at 500 g at 4 °C and resuspended in 500 μL of ice-cold cryopreservation solution (50% FBS, 40% cardiomyocyte growth media, 10% DMSO). 500 μL of cell suspension for each sample was transferred to a 2 mL cryotube on ice. The cryotubes were then transferred to a pre-chilled Mr. Frosty container and placed at -80°C overnight to complete cryopreservation.

∼300,000 cells were shipped to Active Motif to perform an Assay for Transposase-Accessible Chromatin using sequencing (ATAC-seq). The resulting material was quantified by the KAPA Library Quantification Kit for Illumina platforms (KAPA Biosystems), and sequenced with PE42 sequencing on the NextSeq 500 sequencer (Illumina). Cutadapt (3.0) was used to remove the adapters and low-quality sequences from sequencing reads. Cleaned reads were then mapped to the rn4 rat genome reference by Bowtie2 (2.2.7) with the ‘very sensitive’ option and ‘-k’ being set as 10. After alignment, the narrow peaks were called by using MACS2 (2.2.6) with ‘--shift’, ‘--extsize’ being set as -100 and 200, respectively. To compare the ATAC-seq signal between two conditions, MACS2 bdgdiff was used with the recommended settings. Condition-unique peaks were then annotated to genes by Homer (4.8).

The downstream miRNA-mRNA interactions were predicted using rat-specific miRDB (https://mirdb.org/) and Rat Genome (https://rgd.mcw.edu/rgdweb/homepage/). For the quantification of ATAC-seq signal of pre-defined regions, the ‘compute Matrix’ function provided by deep tools (3.5.0) was used. ATAC-seq intensity signal was calculated using a range of -2kb to +2kb region surrounding the transcriptional starting site (TSS) for each gene.

#### AgomiRs

Chemically synthesized AgomiRs (hsa-let-7c-5p, hsa-miR-101-3p, and hsa-miR-499a-5p) were purchased from Applied Biological Materials, Inc.

#### Transfections, RNA extraction, qPCR, and RNA-Seq

Primary human cardiomyocytes were transfected with 2.5mg of SRF, STEMIN, YAP1, YAP5SA, and COMBO (YAP5SA+STEMIN) mRNAs using Lipofectamine™ MessengerMAX™ (ThermoFisher). mRNAs were transfected with 5 ml of MessengerMAX reagent in 6-well extracellular matrix plates (∼9.6cm^2^ growth area per well) at a maximum confluency of 1-1.2 million cells for miRNA/mRNA-seq assay.

mRNA/miRNA libraries were prepared and sequenced at the University of Houston Seq-N-Edit Core per standard protocols. Briefly, mRNA/miRNA samples were extracted using the miRNeasy kit by Qiagen. mRNA/miRNA libraries were prepared with the QIAseq mRNA/miRNA library kit (Qiagen) using 5 μL of the extracted mRNA/miRNA sample. Libraries were produced by sequentially ligating adapters to the 3' and 5' ends of mRNA/miRNAs. This was followed by reverse transcription into cDNA and subsequent ligation of sample indexes and sequencing adapters. The size selection for libraries was performed using QIAseq beads (Qiagen). Library purity was analyzed using the DNA HS1000 tape on a Tapestation 4200 (Agilent) and quantified with a Qubit Fluorometer (Thermo Fisher). The prepared libraries were pooled and sequenced using the NextSeq 2000 (Illumina), generating ∼20 million 76 bp single-end reads per sample.

miRNA data were analyzed using the QIAseq miRNA quantification and analysis pipelines through Gene Globe (Qiagen). Sequencing reads were trimmed for adapters and low-quality bases. The insert sequences and unique molecular indices were then identified, and reads with less than 16 bp insert sequences or less than 10 bp UMI sequences were discarded. Following this, Bowtie was used to map to different databases based on a sequential alignment strategy. Read counts for each RNA category (miRBase mature, miRBase hairpin, piRNA, tRNA, rRNA, mRNA, and other RNA) are calculated from the mapping results. miRBase V21 is used for miRNA, and piRNABank is used for piRNA. Normalization of mRNA/miRNA data was performed using the trimmed mean of M-values.

Differential gene expression quantification was performed using DESeq2 package in R-studio. Differentially expressed genes (DEG) were determined with fold change > 1 and p-adjusted < 0.1, as well as genes with more than one read per kilobase of the transcript, per million mapped reads (RPKM), under the test conditions as compared to the controls. Gene ontology analysis was performed using Bioconductor-cluster Profiler packages. RNA-seq analysis was done at RStudio (3.6.0). Heat maps were plotted using the R “pheatmap” package.

AC16 cells were also transfected with mRNA 0.25mg of STEMIN, YAP5SA, and COMBO (YAP5SA+STEMIN) using 5 ml of MessengerMAX reagent in 6-well plates (∼9.6 cm^2^ growth area per well) at a maximum confluency of 1-1.2 million cells for the qPCR assays, according to the manufacturer’s protocol. 1mg of AgomiRs were also transfected with 7.5ml of RNAiMAX (ThermoFisher) reagent in 6-well plates with the same cell density for the qPCR experiments, according to the manufacturer’s protocol. Primer design was done using IDT-DNA Primer Quest Tool, qPCR was normalized using GAPDH as the housekeeping gene, and analyzed via the ΔΔC_T_ method.

Total RNA was isolated using the Zymo Quick-RNA kit. Reverse transcription for qPCR involved M-MLV reverse transcriptase (QuantaBio qScript cDNA SuperMix), Poly(A) polymerase, ATP, and dNTPs (NEB). qPCR was performed using SYBR Green Master Mix (Applied Biosystems™) on an Agilent AriaMx real-time PCR system.

#### Transfection efficiency was determined in all cases by western blot

For the Luciferase experiments, 1mg of the plasmid was co-transfected with 1mg of AgomiRs in 6-well plates (∼9.6cm^2^ growth area per well) to AC16 cells at a maximum confluency of 1-1.2 million cells. AgomiRs were transfected with 7.5ml of RNAiMAX (ThermoFisher) reagent according to the manufacturer’s protocol. 1mg of Firefly-3’-UTR and Renilla luciferase constructs were delivered using jetPRIME® transfection reagent (PolyPlus Transfection SA) overnight, after which 1mg of AgomiRs was transfected and allowed to stay for 12 hours.

#### Exosome Isolation and transfection

AC16 cells were cultured in 10 cm dishes and transfected every 24 hours for three days (up to 72 h) with 10 μg of either STEMIN or YAP5SA mRNA using Lipofectamine™ MessengerMAX™. Control cells received empty reagent. Conditioned media (10 mL per collection, totaling 30 mL) were pooled, centrifuged at 3000 rpm for 1 hour, and supernatants were stored at −80°C. Exosomes were then isolated using the Qiagen Exo-easy Maxi kit via spin-column purification and characterized via nanoparticle tracking analysis for size and concentration. Exosomal miRNA-seq was conducted at UH-SNEC using the Illumina NextSeq 2000 (Illumina).

#### Staurosporine-induced apoptosis assay

AC16 cells were seeded into a 3-well removable chamber at 0.1-0.12 million cells (∼1.6cm^2^ growth area per well) and cultured for 24 h. Cells were transfected with either 0.25 or 0.50 μg of STEMIN or YAP5SA modRNA, or with 0.50 μg AgomiRs (hsa-miR-101-3p, hsa-miR-499a-5p, or hsa-let-7c-5p). Controls included untreated and staurosporine-treated cells transfected with Lipofectamine alone. After 24 h, all experimental and positive control groups were treated with 1 μM staurosporine (MedChem Express, 98.89% purity) for 3 h to induce apoptosis.

#### Immunofluorescence and imaging

For immunofluorescence assays, AC16 cells were cultured in a 3-well Ibidi chamber, fixed, permeabilized, and stained using cleaved Caspase-3 primary antibodies (Cell Signaling Technology) and Alexa Fluor™ 488 secondary antibodies, along with Click-iT Plus TUNEL assay reagents (ThermoFisher). Imaging was performed on a Nikon Ti-E inverted microscope equipped with a DS-U3 5 MP color camera. Fluorescence intensities (FITC-Alexa Fluor 488, Alexa Fluor 594 for TUNEL, and DAPI) were analyzed using ImageJ histogram tools.

#### Flow cytometry

Cells were strained through 35 μm nylon mesh (Sartorius) into 12×75 mm flow tubes (Corning), stained with CellEvent™ Caspase-3/7 Green Detection Reagent (ThermoFisher), and analyzed on a BD Accuri™ C6 Plus flow cytometer. For exosome treatments, AC16 cells were cultured for 24 h, incubated with control-, STEMIN-, or YAP5SA-derived exosomes in serum-free media, then treated with 2.0 μM staurosporine for 6 h before staining and analysis. These experiments were conducted in triplicate and analyzed using Floreada (https://floreada.io/), which implements automatic compensation using the AutoSpill algorithm. AutoSpill is a novel method for calculating compensation matrix.

#### Immunofluorescence and imaging (exosome experiments)

AC16 cells were seeded into a 3-well removable chamber at 0.1-0.12 million cells (∼1.6cm^2^ growth area per well), and cultured in standard media for 24 h, after which they were refreshed with serum-free, cell-free media, and incubated with purified exosomes from control, STEMIN, or YAP5SA-transfected cells. After 6 h of 2.0 μM staurosporine treatment, cells were fixed, permeabilized, and immune-stained as described above. Imaging was conducted using a Nikon Ti-E microscope with a DS-Fi1 5 MP camera, and fluorescence (FITC, AF594, and DAPI) was quantified with ImageJ.

The reason for increasing the duration from 3 to 6 hours of exposure to staurosporine, and its concentration from 1 to 2 μM for the exosomes experiments, was to check if we could observe a more drastic apoptotic phenotype and recovery with the treatment, but they ended up yielding comparable results when compared to the control.

#### Dual-luciferase reporter assay

3’-UTRs of CDKN1A, RUNX1, SOX6, CASP3, RB1, TP53, and PDCD4 were cloned downstream of plasmid cDNA coding for Firefly Luciferase (FIRE (Addgene plasmid #18964)), using restriction cloning and validated by Sanger sequencing. The resulting FIRE-3’-UTRs (1.0-μg) were then co-transfected with Renilla Luciferase (1.0-μg), in AC16 Cardiomyocytes previously cultured and stabilized in regular media, using the jetPRIME® transfection reagent.

After 4 hours, the media was refreshed, and the cells were transfected with 1.0 μg of AgomiRs targeting hsa-miR-101-3p, hsa-miR-499a-5p, and hsa-let-7c-5p. 24 hours later, the cells were harvested, total cellular protein was extracted with Passive Lysis 5X Buffer (Promega), and the dual luciferase reporter (DLR) assay was performed on the samples using the Dual-Glo® Luciferase Assay System (Promega) on a BioTek Synergy 2 plate reader. The resulting data was organized into Relative Luminescence (Differential Luciferase (Firefly/Renilla) Inhibition) plots.

#### Tissue collection and histology

The hearts were then fixed in paraffin wax, and 5μm tissue sections from them were obtained on glass slides; these sections were deparaffinized, immuno-stained with cleaved-Caspase-3 primary antibody (Cell Signaling Technology Cat#9661), Alexa Fluor™ 488 (ThermoFisher Scientific) secondary antibody, and mounted with ibidi Mounting Medium plus DAPI. The slides were cured for 48 hours, and immunofluorescence images were obtained using a Nikon Ti-E inverted microscope equipped with a DS-Fi1 5-megapixel color camera (Nikon Instruments). The images were then analyzed using ImageJ.

### Quantification and statistical analysis

#### Statistical analyses

##### Welch test

Welch’s t-test was used for pairwise comparisons between groups to account for potential differences in variance and sample size. This approach estimates group-specific variances and applies a correction to the degrees of freedom. All statistical tests were two-sided, with *p* < 0.05 considered statistically significant. Welch test was used to analyze data in Figures 4G, S2A–S2E, and S3C–S3F. Data are presented as mean ± SEM. Data in Figure 4G was done on 7 mice (3 control (2 males, 1 female) and 4 experimental (2 males, 2 females)), while the ones in Figures S2A–S2E and S3C–S3F were done in triplicate (n=3), 3 wells of AC16 cells in 6-well plates at 80-85% confluency (0.96 – 1 million cells).

##### Analysis of variance

Analysis of variance (ANOVA) was used for comparisons among three or more groups. All tests were two-sided, and statistical significance was defined as *p* < 0.05. Data are presented as mean ± SEM. ANOVA was used to analyze data in Figures 4B, 4D, 5B, and 5C. These experiments were done in triplicate (n=3), 3 wells of AC16 cells in 3-well chamber, removable (Ibidi) at 80-85% confluency (0.096 – 0.100 million cells).

##### Power Analysis

Power Analysis on the Immunohistochemistry assay in Figures 4E–4G. Power analysis indicates that, with seven animals, the study achieves an Estimated Power of 0.8184, sufficient to detect the observed effect at an Alpha Level of 0.05.

**Table:** 

N = 7, Alpha = 0.05
N = 7
N1 = 3
N2 = 4
m1 = 0.8980
m2 = 0.6120
sd1 = 0.0944
sd2 = 0.1140
Code: power two means 0.898 0.612, n1(3) n2(4) sd1(0.0944) sd2(0.1140) alpha(0.05).
Estimated power: 0.8184

### Additional resources

Statistical details of experiments, including the statistical tests and analyses used, replicates, graphs, & charts, definition of center, and dispersion & precision measures (e.g., mean, SD, SEM, confidence intervals), used for this study can be found in the “Data S1” file attached to this submission (as a supplemental file), and also at https://doi.org/10.17632/xwm3n5zj4n.1.

## Supplemental information

Document S1. Figures S1–S5 and Tables S1 and S2

Data S1. Data, tables, charts and analyses related to 1D-G, 4B, D, G, 5B-C, S2A-E, and S3A-I in Figures

Data S2. Sequences used in this study
