# Supplementary material for: STEMIN- and YAP5SA-induced exosomes prevent cardiomyocyte apoptosis
Source: iScience. 2026 Jun 22;29(7):116277. doi: 10.1016/j.isci.2026.116277 (PMC13316227; doi:10.1016/j.isci.2026.116277)
Supplement: Document S1. Figures S1–S5 and Tables S1 and S2 [file mmc1.pdf]

iScience, Volume *29*

Supplemental information

STEMIN- and YAP5SA-induced exosomes prevent cardiomyocyte apoptosis

Adeniyi Adeleye, Siyu Xiao, Ilkin Tetik Altintop, Azeez Muili, Micah Castillo, Preethi Gunaratne, Kacie Waiters, Tasneem Bawa-Khalfe, Bradley K. McConnell, and Robert J. Schwartz

SUPPLEMENTAL FIGURES AND TABLES

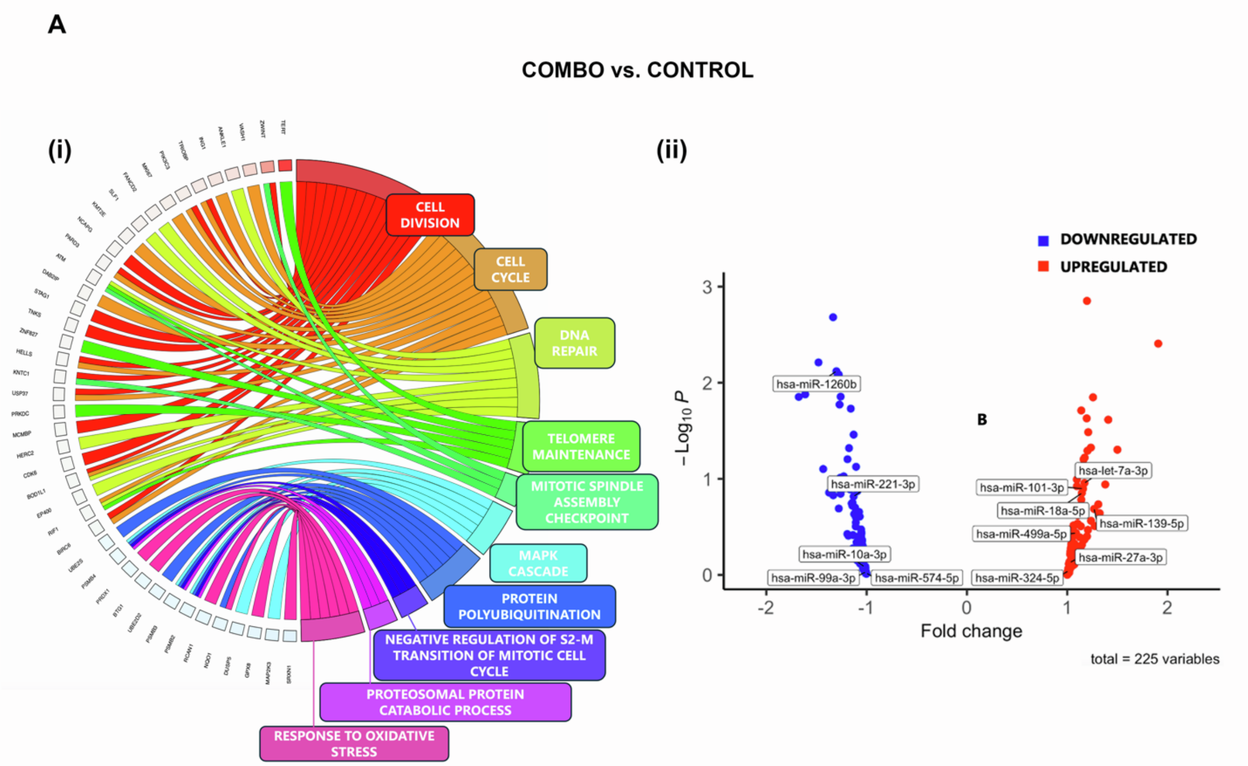

(iii)

TOP 29 GENES UPREGULATED IN COMBO-TREATED CARDIOMYOCYTES									
TERT	ZWINT	VASH1	ANKLE1	ING1	TRIOBP	PIK3C3	MKI67	FANCD2	SLF1
KMT2E	NCAPG	PARD3	ATM	DAB2IP	STAG1	TNKS	ZNF827	HELLS	KNTC1
USP37	PRKDC	MCMBP	HERC2	CDK6	BOD1L1	EP400	RIF1	BIRC6	
TOP13 GENES DOWNREGULATED IN COMBO-TREATED CARDIOMYOCYTES									
UBE2S	PSMB4	PRDX1	BTG1	UBE2D2	PSMB3	PSMB2	RCAN1	NQO1	DUSP5
GPX8	MAP2K3	SRXN1							

Supplemental Figure S1. Differentially-expressed Gene Ontology/Phenotypes in COMBO-treated primary cardiomyocytes.
(A(i)) Gene Ontology chord plot of differentially upregulated and downregulated phenotypes (GO-terms),
(A(ii)) miRNAs differentially expressed in COMBO-treated cardiomyocytes, and
(A(iii)) top differentially expressed genes (downregulated and upregulated) by COMBO.

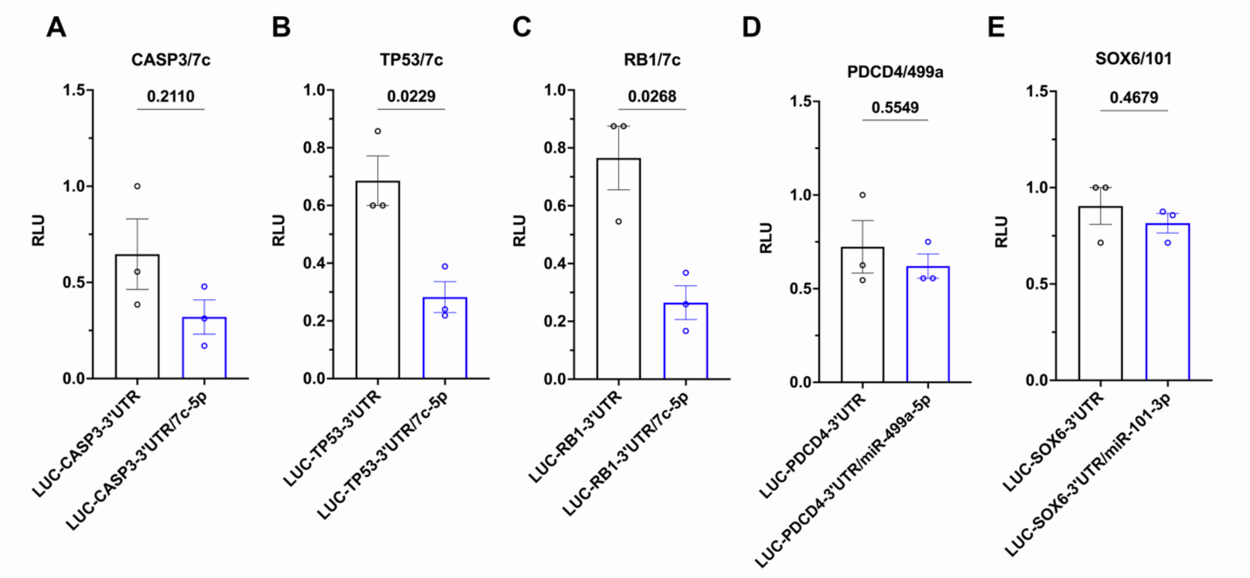

Supplemental Figure S2. Selected miRs hsa-miR-101-3p, hsa-miR-499a-5p, and hsa-let-7c-5p target the 3'-UTRs of apoptotic and anti-survival markers. Differential luminescence plots from dual luciferase reporter assay:

(A-E), showing varying degrees of inhibition of luciferase activity in FIRE(firefly)-3'-UTRs by miRNAs: there was inhibited expression of luciferase in:

(A) FIRE-CASP3-3'-UTR,

(B) FIRE-TP53-3'-UTR, and

(C) FIRE-RB1-3'-UTR by let-7c-5p,

in

(D) FIRE-PDCD4-3'-UTR by miR-499a-5p, and

in

(E) FIRE-SOX6-3'-UTR by miR-101-3p.

Data are represented as the mean (p-value), and analyzed by Welch test.

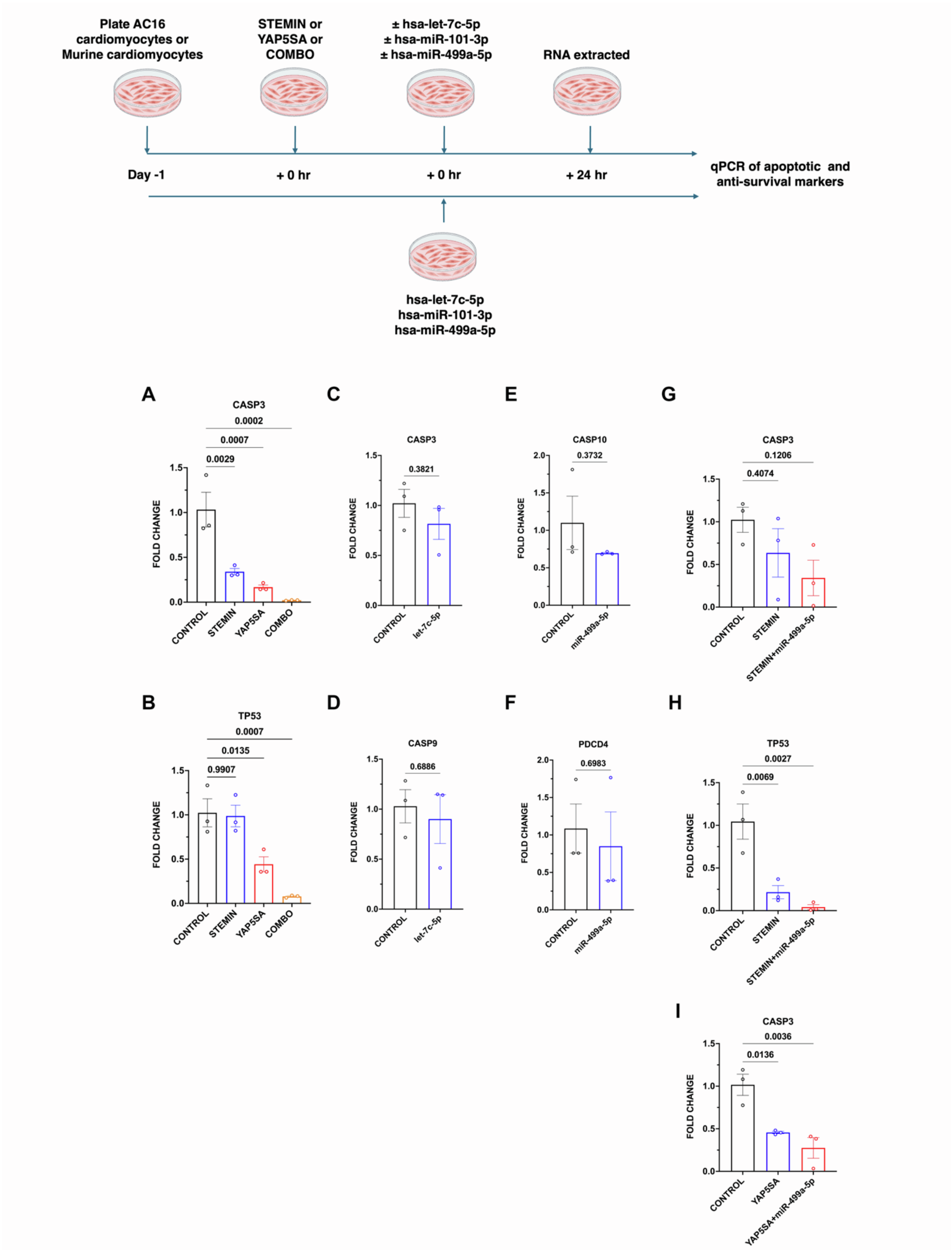

Supplemental Figure S3: STEMIN, YAP5SA, and their induced anti-apoptotic microRNAs in cardiomyocytes downregulate apoptotic gene activity. A schematic diagram shows the experimental protocol to determine the differential (qPCR) expression profiles of (A-I):

(A) CASP3 and

(B) TP53, in STEMIN-, YAP5SA- and COMBO-treated AC16 cardiomyocytes;
of:
(C) CASP3 and
(D) CASP9 in let-7c-5p-treated cardiomyocytes;
of:
(E) CASP10 and
(F) PDCD4 in miR-499a-5p-treated cardiomyocytes;
of:
(G) CASP3 and
(H) TP53 in STEMIN±miR-499a-5p-treated primary mouse cardiomyocytes; and
of:
(I) CASP3 in YAP5SA±miR-499a-5p-treated primary mouse cardiomyocytes cultured ex-vivo.
Data in A, B, G, H, and I are represented as mean (p-value), analyzed by one-way ANOVA.
Data in C-F are represented as mean (p-value), and analyzed by Welch test.

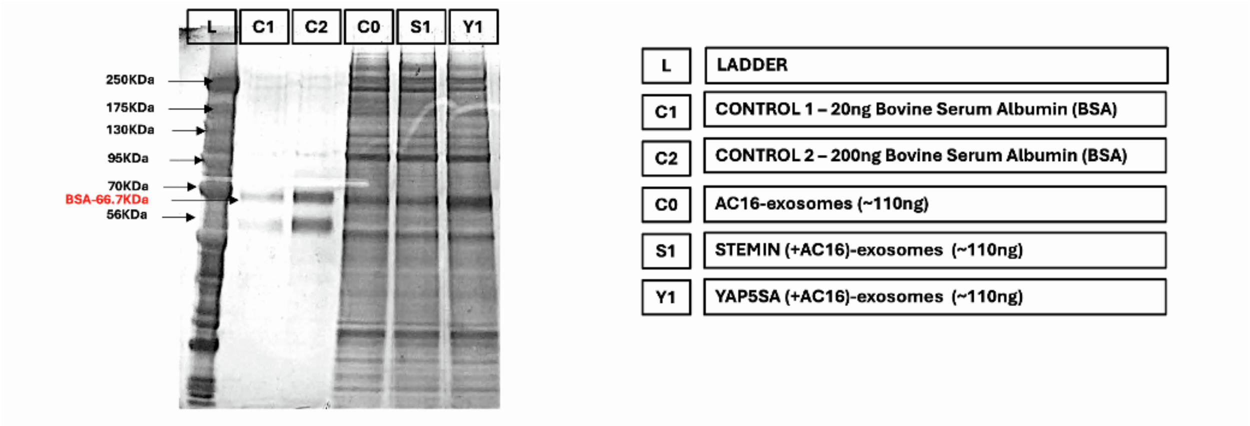

Supplemental Figure S4. **Characterization and Estimation of proteins extracted from exosomes**. Silver-stained images of total protein extracted from exosomes (20ul) of each sample, estimated to contain 275 ng of protein, run on PAGE. Molecular weight standards are shown on lane L.

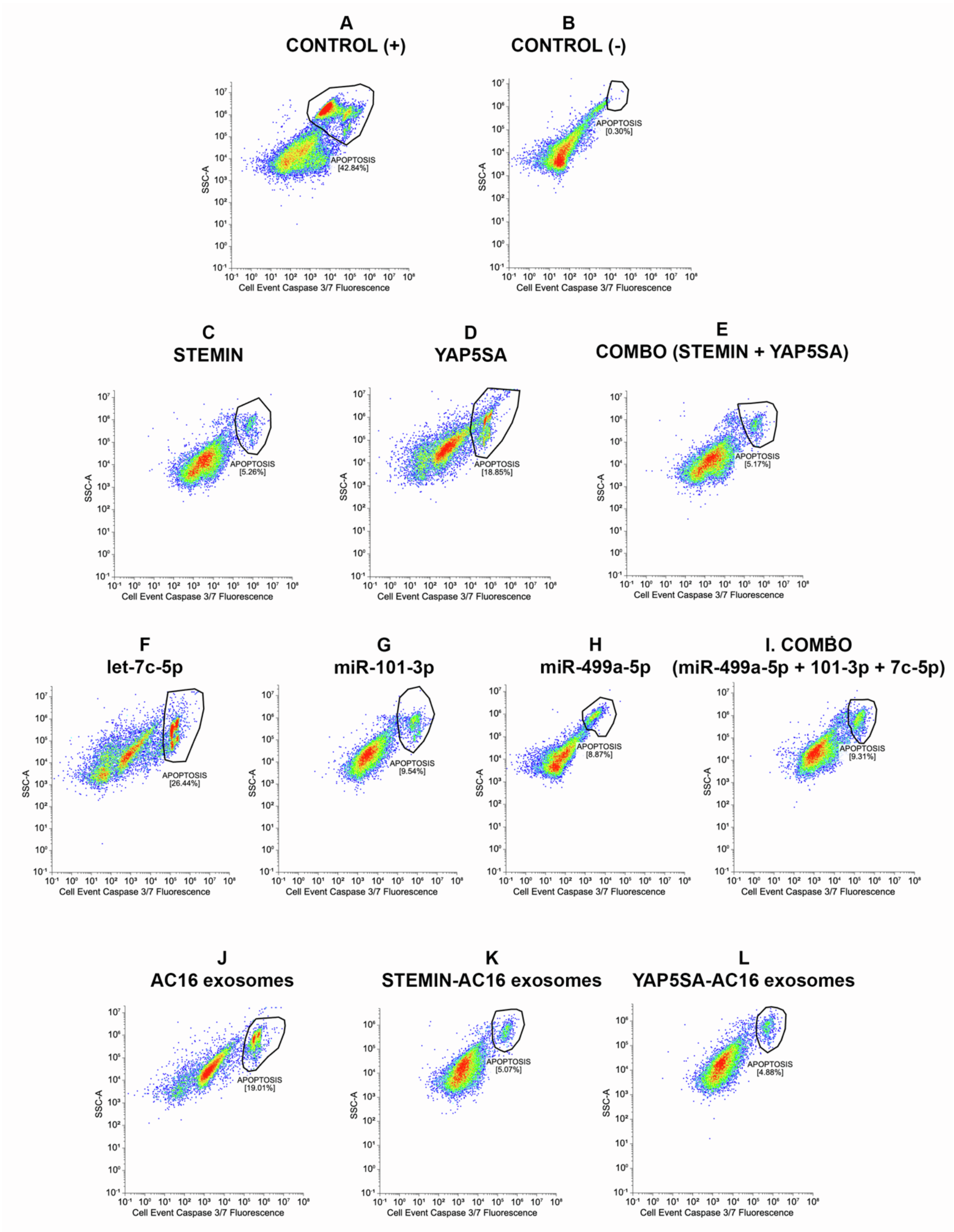

Supplemental Figure S5 (A-E). Flow Cytometric Estimation of Apoptosis in modRNA (A-E), miRNA (F-I), and exosome-treated (J-L) Cardiomyocytes. Flow cytometric plots (A-L), showing apoptotic events in AC16 cardiomyocytes transfected with (all including staurosporine, except the negative control, B):
(A) empty reagent, serving as a positive control,

(B) empty reagent (no staurosporine), serving as a negative control,
(C) 1.0 µg mRNA of STEMIN (mutant SRF),
(D) 1.0 µg mRNA YAP5SA (mutant YAP1),
(E) COMBO (0.5µg mRNA of STEMIN+0.5 µg mRNA YAP5SA),
(F-I) 1.0 µg AgomiRs of:
(F) hsa-let-7c-5p,
(G) hsa-miR-101-3p,
(H) hsa-miR-499a-5p, and
(I) COMBO miR (0.33µg hsa-let-7c-5p+ 0.33µg hsa-miR-101-3p + 0.33µg hsa-miR-499a-5p).
(J-L) exofected with:
(J) 10x-AC16-only-exosomes (control),
(K) 10x-STEMIN- and
(L) 10x-YAP5SA-generated exosomes,
containing 275ng total exosomal protein extract estimated from a Silver Staining assay.

TABLE S1: List of oligos used to construct Luciferase-3'-UTR

OLIGO NAME	SEQUENCE
PDCD4-3-UTR-sense	AGGAGATGGAGGTCTCTAGAAGAGAGCTACTGAATATAAGAACTCTTGCAGTCTTAGATGTTATAAAAATATATATCTGAGGGCCCTTGTTAGCACAAGT
PDCD4-3-UTR-antisense	ACTTGTGCTAACAAGGGCCCTCAGATATATATTTTTATAACATCTAAGACTGCAAGAGTTCTTATATTCAGTAGCTCTCTTCTAGAGACCTCCATCTCCT
CDKN1A-3-UTR-sense	TCGGCTCCCCATGTGTCTAGATCCTGGTTCCCGTTTCTCCACCTAGACTGTAAACCTCTCGAGGGCAGGGACCACACCGGGCCCCTGTACTGTTCTGT
CDKN1A-3-UTR-antisense	ACAGAACAGTACAGGGGCCCGGTGTGGTCCCTGCCCTCGAGAGGTTTACAGTCTAGGTGGAGAAACGGGAACCAGGATCTAGACACATGGGGAGCCGA
RUNX1-3-UTR-sense	TTTTTTGAGAAACTCTAGAAGCTACAGCTTTGGGTCATTTTTAACTACTGTATTCCCACAAGGAATCCCCAGATATTTAGGGCCCTGTGTTGATAATTT
RUNX1-3-UTR-antisense	AAATTATCAACACAGGGCCCTAAATATCTGGGGATTCCTTGTGGGAATACAGTAGTTAAAAATGACCCAAAGCTGTAGCTTCTAGAGTTTCTCAAAAAAA
SOX6-3-UTR-sense	TACCTTAGACAAGCTTTCTAGAATAAGCTGTTACTACATAACTTATCTTACTGTAACTCTTTTATTTCCCCCGACGTTGTAGGGCCCATTTGTTTGTGATG
SOX6-3-UTR-antisense	CATCACAAACAAATGGGCCCTACAACGTCGGGGGAAATAAAAGAGTTACAGTAAGATAAGTTATGTAGTAACAGCTTATTCTAGAAAGCTTGTCTAAGGTA
RB1-3-UTR-sense	TATAACCATATGATTCTAGAACTATCATACTACTGAAACAGATTTCATACCTCAGAATGTAAAAGAACTTACTGATTATTGGGCCCTTCTTCATCCAACT
RB1-3-UTR-antisense	AGTTGGATGAAGAAGGGCCCAATAATCAGTAAGTTCTTTTACATTCTGAGGTATGAAATCTGTTTCAGTAGTATGATAGTTCTAGAATCATATGGTTATA
CASP3-3-UTR-sense	TTAAGACATACTCCTCTAGATTCCATCAAATAGAACCACTATGAAGCTACCTCAAACTTCCAGTCAGGTAGTTGCAATTGGGGCCCAATTAAATTAGGAA
CASP3-3-UTR-antisense	TTCCTAATTTAATTGGGCCCCAATTGCAACTACCTGACTGGAAGTTTGAGGTAGCTTCATAGTGGTTCTATTTGATGGAATCTAGAGGAGTATGTCTTAA
TP53-3-UTR-sense	AGGGCTCACTCCAGTCTAGACCACCTGAAGTCCAAAAAGGGTCAGTCTACCTCCCGCCATAAAAAACTCATGTTCAAGACGGGCCCAGAAGGGCCTGACT
TP53-3-UTR-antisense	AGTCAGGCCCTTCTGGGCCCGTCTTGAACATGAGTTTTTTATGGCGGGAGGTAGACTGACCCTTTTTGGACTTCAGGTGGTCTAGACTGGAGTGAGCCCT

Table S2: miRNA profiles to known exosomal sequence motifs

miRNA Symbol	Mature Sequence	# EXO-motifs in Sequence	EXO-motif (with count of miRNAs)
hsa-let-7c-5p	UGAGGUAGUAGGUUGUAUAGUU	1	UGAG (6)
hsa-let-7f-5p	UGAGGUAGUAGAUUGUAUAGUU	1	UGUG (4)
hsa-let-7i-5p	UGAGGUAGUAGUUUGUGCUGUU	2	CAGU (3)
hsa-miR-10b-5p	UACCCUGUAGAACCGAAUUUGUG	2	GGAG (2)
hsa-miR-122-5p	UGGAGUGUGACAAUGGUGUUUG	4	CCCU (2)
hsa-miR-1285-3p	UCUGGGCCAAAGGAGGACCCU	1	GGCG (2)
hsa-miR-148b-3p	UCAGUGCAUCACAGAACUUGGU	1	UCCU (2)
hsa-miR-155-5p	UUAAUGCUAAUCGUGAUAGGGGUU	0	GGCC (1)
hsa-miR-15a-5p	UAGCAGCACAUAAUGGUUUGUG	1	UGAC (1)
hsa-miR-16-5p	UAGCAGCACGUAAAUAUUGGCG	1	CAUU (1)
hsa-miR-17-5p	CAAAGUGCUUACAGUGCAGGUAG	1	CCCA (0)
hsa-miR-186-5p	CAAAGAAUUCUCCUUUUGGGCU	1	CCCG (0)
hsa-miR-196a-5p	UAGGUAGUUUCAUGUUGUUGGG	0	CCGA (0)
hsa-miR-21-5p	UAGCUUAUCAGACUGAUGUUGA	0	GCCG (0)
hsa-miR-214-3p	ACAGCAGGCACAGACAGGCAGU	1	GGUC (0)
hsa-miR-26b-3p	UUCAAGUAAUUCAGGAUAGGU	0	UGUC (0)
hsa-miR-26b-5p	UCCAUAGGAGAUUUCAGGGA	0	GGAC (0)
hsa-miR-30a-5p	UGUAAACAUCCUCGACUGGAAG	1	UCCG (0)
hsa-miR-335-5p	UCAAGAGCAAUAACGAAAAAUGU	0	UGCG (0)
hsa-miR-382-5p	AAGUUGUCCUUCGUCCCGAG	0	UCCA (0)
hsa-miR-421	ACAUACACAGCAUAAUAAUGGCG	2	UGCC (0)
hsa-miR-665	ACAGGAGGCUGCGGC CCU	4	GCCU (0)
